# Intra-cluster and inter-period correlation coefficients for cross-sectional cluster randomised controlled trials for type-2 diabetes in UK primary care

**DOI:** 10.1186/s13063-016-1532-9

**Published:** 2016-08-15

**Authors:** James Martin, Alan Girling, Krishnarajah Nirantharakumar, Ronan Ryan, Tom Marshall, Karla Hemming

**Affiliations:** Institute of Applied Health Research, University of Birmingham, Birmingham, B15 2TT UK

**Keywords:** Intra-cluster correlation coefficient, Inter-period correlation coefficient, Cluster randomised trial, Type-2 diabetes

## Abstract

**Background:**

Clustered randomised controlled trials (CRCTs) are increasingly common in primary care. Outcomes within the same cluster tend to be correlated with one another. In sample size calculations, estimates of the intra-cluster correlation coefficient (ICC) are needed to allow for this nonindependence. In studies with observations over more than one time period, estimates of the inter-period correlation (IPC) and the within-period correlation (WPC) are also needed.

**Methods:**

This is a retrospective cross-sectional study of all patients aged 18 or over with a diagnosis of type-2 diabetes, from The Health Improvement Network (THIN) database, between 1 October 2007 and 31 March 2010. We report estimates of the ICC, IPC, and WPC for typical outcomes using unadjusted and adjusted generalised linear mixed models with cluster and cluster by period random effects. For binary outcomes we report on the proportions scale, which is the appropriate scale for trial design. Estimated ICCs were compared to those reported from a systematic search of CRCTs undertaken in primary care in the UK in type-2 diabetes.

**Results:**

Data from 430 general practices, with a median [IQR] number of diabetics per practice of 241 [150–351], were analysed. The ICC for HbA1c was 0.032 (95 % CI 0.026–0.038). For a two-period (each of 12 months) design, the WPC for HbA1c was 0.035 (95 % CI 0.030–0.040) and the IPC was 0.019 (95 % CI 0.014–0.026). The difference between the WPC and the IPC indicates a decay of correlation over time. Following dichotomisation at 7.5 %, the ICC for HbA1c was 0.026 (95 % CI 0.022–0.030). ICCs for other clinical measurements and clinical outcomes are presented. A systematic search of ICCs used in the design of CRCTs involving type-2 diabetes with HbA1c (undichotomised) as the outcome found that published trials tended to use more conservative ICC values (median 0.047, IQR 0.047–0.050) than those reported here.

**Conclusions:**

These estimates of ICCs, IPCs, and WPCs for a variety of outcomes commonly used in diabetes trials can be useful for the design of CRCTs. In studies with observations taken at different time-points, the correlation of observations may decay over time, as reflected in lower values for the IPC than for the ICC. The IPC and WPC estimates are the first reported for UK primary care data.

**Electronic supplementary material:**

The online version of this article (doi:10.1186/s13063-016-1532-9) contains supplementary material, which is available to authorized users.

## Background

Diabetes is an important public health issue [1] and an increasing number of clinical trials are being conducted to improve care for patients with diabetes. Increasingly, interventions aimed at improving the quality of care are evaluated using cluster randomised controlled trials (CRCTs) [2–5]. Whilst observations used in the evaluation may still be made at the individual level, randomisation at the cluster level (such as GP surgery) will often be necessary [5–7] and is increasingly being used [8]. In CRCTs patients within the same cluster tend to more similar than patients from differing clusters [7, 9]. Thus, the observations within a cluster may not be independent, and the design and analysis of CRCTs should acknowledge this [5, 10–13].

Important outcomes in trials of diabetes include clinical measurements, such as glycosylated haemoglobin (HbA1c) (both as a continuous and dichotomised outcome) [14], body mass index (BMI) [15], cholesterol [16], blood pressure [17], or the incidence of macrovascular and microvascular outcomes [18, 19].

Sample size calculations for an individually randomised controlled trial (RCT) are relatively straightforward, but for a CRCT it is necessary to account for the nonindependence [10–12]. A design effect can be used to inflate the sample size of an RCT to that required in a CRCT [9, 20]. For a trial with equal cluster sizes, the design effect is calculated as:

1 $$ 1+\left(m-1\right)\rho . $$

Here *m* is the cluster size and *ρ* is the correlation between patients within a cluster [21]. This correlation has important implications for the sample size required [22, 23].

The majority of CRCTs have a parallel design. That is to say, clusters are allocated to either intervention or control. However, increasingly, the value of alternative cluster designs is being appreciated. Some alternative designs include the cluster cross-over [24], the stepped wedge [25, 26], and the dog-leg [27, 28]. In these alternative designs repeated cross-sectional samples are taken from each cluster over multiple time periods. It is becoming increasingly recognised that observations from the same cluster and same period are likely to be more highly correlated than observations in the same cluster but at different periods [29–32]. This leads to the notion of a within-period cluster correlation (WPC) and an inter-period cluster correlation (IPC). Unfortunately, there is little or no empirical literature to inform likely values for these parameters at the design stage [28, 29].

For a trial to be powered correctly, an accurate estimate of the correlation of observations within a cluster is required. In the past, many type-2 diabetes trials in primary care have failed to report this correlation, forcing many planned trials to use ad hoc values at the design stage [33]. This leads to inaccurate sample size estimates and (sometimes) to underpowered trials. Typically, this correlation is assumed to be time independent – and a single intra-cluster correlation coefficient (ICC) is used in the sample size calculation. This assumption may not always be valid. For designs with observations taken over multiple time periods, estimates of the WPC and IPC are vital in the sample size calculation [28, 29]. These can be obtained from routinely collected data, in a similar way to ordinary ICCs [34, 35].

Our objective here is to estimate ICCs for typical trial outcomes related to type-2 diabetes using anonymised patient data from The Health Improvement Network database [36]. We additionally report estimates of the WPC and the IPC for a subset of continuous outcomes. Finally, we review previous CRCTs in type-2 diabetes to compare the ICCs estimated in this paper to those previously used.

## Methods

### Correlation of observations in a cluster trial

The quantity *ρ* in Eq. 1 is defined as the correlation between two randomly selected observations within the same cluster. Typically, an assumption is made that this correlation is independent of the timing of the observations. This property is consistent with a decomposition of the total variance into two independent components representing variation between clusters and between subjects (within clusters). In view of this, the ICC can be defined as the proportion of the variance that is attributable to the between-cluster variance, given as:

2 $$ \frac{{\sigma_b}^2}{{\sigma_b}^2+{\sigma_w}^2}, $$

where *σ*_*b*_^2^ and *σ*_*w*_^2^ represent the between- and within-cluster variance components.

Cluster trials are typically analysed using a multilevel linear model. If the correlation between observations in a cluster is independent of when they are taken, an approach using the ratio of variances is a simple method to estimate the ICC. This approach is taken throughout the paper whenever an estimated ICC is reported.

### Time-dependent correlation

In some contexts, a model based on the assumption of time-independent correlations is flawed. An alternative model can be fitted to the data by splitting time into a number of (equal) periods. In this formulation, constant correlations are assumed: (1) for any two observations in the cluster from the same time period (WPC); and (2) for any two observations from the same cluster in different time periods (IPC).

These assumptions are consistent with a variance-decomposition into three independent components: between clusters (*σ*_*e*_^2^); between time periods (within clusters) (*σ*_*c*_^2^); and between subjects (within time period and cluster) (*σ*_*t*_^2^).

Now, the WPC is the correlation of observations between two patients in the same cluster from the same time period. This can be calculated as:

$$ \frac{{\sigma_c}^2+{\sigma_t}^2}{{\sigma_c}^2+{\sigma_e}^2+{\sigma_t}^2}. $$

The IPC is the correlation of observations between two patients in the cluster from different time periods, and is calculated as:

$$ \frac{{\sigma_c}^2}{{\sigma_c}^2+{\sigma_e}^2+{\sigma_t}^2}. $$

In this framework, the correlation, *ρ*, between two randomly selected observations within the same cluster is given by a within-cluster correlation (WCC) defined by:

3 $$ WCC=IPC+\frac{1}{n_{tp}}\left(WPC-IPC\right). $$

Here *n*_*tp*_ is the number of time periods in the study. It is assumed that each time period contains an equal number of observations.

The ratio of the IPC to the WPC is known as the cluster autocorrelation (CA), which is the correlation between the cluster level mean outcome over time [28]. The cluster autocorrelation has been established as key to sample size formula for studies with a repeated cross-sectional design [37]. We present estimates of the CA alongside the IPC and WPC.

In the absence of period effects, the CA = 1, indicating that the time-dependent model is unnecessary. In this setting, WCC = WPC = IPC. Otherwise it follows from the definitions that WPC > WCC > IPC.

### Correlation of binary outcomes

In the context of a clinical trial, data are often dichotomous – recording the presence or absence of a particular clinical outcome. The ICC that appears in the design effect is then defined as the correlation between two binary outcomes from two patients in the same cluster. In such cases, sample size calculations will typically entail a normal approximation to the binomial distribution which describes the number of positive outcomes in a sample of fixed size. Nevertheless the analysis of dichotomous outcomes in cluster trials is often conducted via a multilevel logistic model. In such models the observed binary outcome may be conceptualised as having arisen by dichotomising a continuous latent scale. When these models are fitted in some analysis packages (e.g. Stata) a type of ICC is presented which relates not to the observed binary outcomes but to this unobservable latent scale. It takes the form:

$$ \frac{{\sigma_b}^2}{{\sigma_b}^2+{\pi}^2/3}, $$

where *σ*_*b*_^2^ is the between-cluster component of variance on the latent scale and the term *π*^2^/3 is associated with the logistic distribution used to generate the binary model.

Since this version of the ICC refers to the unobservable latent scale, rather than the correlation between the binary outcomes of two patients from within the same cluster, this ICC should not be used directly to compute design effects for sample size calculations. In principle, a latent ICC from a logistic regression model can be converted to a natural ICC on the proportion scale for the raw binary data, taking account of the prevalence of the outcome – see, for example, the table presented by Eldridge et al. [21]. Throughout this paper we maintain the distinction between a natural ICC on the proportion scale and a latent ICC for binary data. It is the natural ICC on the proportion scale that contributes to the calculation of design effects.

### Outcome variables

The aim was to investigate the correlation of all routinely recorded variables that might be clinically relevant to a trial undertaken in type-2 diabetes. The outcome variables were divided into three categories: clinical measures, medication, and clinical outcomes. Clinical measures included HbA1c, systolic blood pressure, diastolic blood pressure, BMI, total cholesterol level, and high-density lipoprotein (HDL) cholesterol level. Medication measurements involved insulin and other hypoglycaemic medications. The clinical outcomes were a first diagnosis of: atrial fibrillation, chronic kidney disease, chronic obstructive pulmonary disease (COPD), ischaemic heart disease (IHD), peripheral vascular disease, and stroke. Patients who had suffered an event prior to the study were excluded from the analysis for that outcome.

### Dichotomisation of continuous outcomes

In practice, many trials use dichotomised values of continuous outcome measures [38, 39], and so we generated dichotomised values for each continuous outcome. A threshold value of 7.5 % was chosen for HbA1c as NICE guidelines state that 7.5 % indicates inadequate control [40], in addition to being used in previous studies [41]. Multiple recommendations have been made that total cholesterol levels should be below 4.0 mmol/L and HDL cholesterol levels be above 1.2 mmol/L [42, 43]. Two relevant cut-points were used for both systolic blood pressure and BMI. For systolic blood pressure, a value of 140 mmHg is the upper limit recommended for patients with type-2 diabetes [40]. A lower value of 130 mmHg is the target that health care professionals aim to reduce systolic blood pressure to in patients who suffer from kidney and eye problems, or those who have suffered a stroke [40]. Two cut-points were chosen for BMI to correspond to the categories of overweight (25 kg/m^2^) and moderately obese (30 kg/m^2^).

### Measurement periods

A cross-sectional sample of measurements taken over a 15-month period was used (1 January 2009 to 31 March 2010), to reflect the NICE quality and outcomes framework (QOF) [44], which monitors measurements taken for patients over a 15-month period. To estimate the IPC and WPC an additional 15 months (1 October 2007 to 31 December 2008) of data is used to estimate the time-dependent correlation, creating two 15-month time periods.

Since the measuring unit of HbA1c changed in 2009 from % to mmol/mol, the consistency in reporting is likely to be poor around this time. In view of this, we consider a slight variation, and a cross-sectional sample of measurements taken over a 12-month period was used (1 January 2008 to 31 December 2008). An additional 12 months (1 January 2007 to 31 December 2007) of data contributes towards the estimation of the IPC and WPC.

### The Health Improvement Network

The retrospective cross-section of patients with type-2 diabetes was formed using data from The Health Improvement Network (THIN) database [36]. Participating general practices contributed anonymised demographics, prescribing information, and clinical data for more than 3.7 million patients throughout the UK. All practices used the Vision computer system.

All patients over 18 years of age were included if a diagnosis of type-2 diabetes, indicated by the appropriate ‘Read codes’, was made before the study index date. Read codes are a coded thesaurus of clinical terms that are used in the recording of patient data in primary care electronic medical records in the UK. The general practices were required to have been using the Vision computer system for a minimum of a 1 year period prior to the study index date, and to have an acceptable mortality reporting (AMR) date (an indicator of practice quality) [45].

### Data summary

The included population was summarised by describing both patient and practice characteristics using appropriate summary statistics. General practice characteristics include the total number of practices, location (country) of the practice, and practice inclusion size (the number of patients from each practice satisfying the entry criteria). Patient characteristics (of the included population) were age (years), gender, location (country of residence), and deprivation quintiles.

We also summarised potential trial outcomes using suitable summary statistics. Outcomes included clinical measures, onset of clinical outcomes, and the prescription of medication. Although the HbA1c variable exhibits skewness, both mean and median values were given as it is assumed to be normally distributed in many trials.

Variation across practices in mean (or median) clinical measures, clinical outcomes, and the prescription of medication, was summarised by reporting the interquartile range (IQR) of the practice mean (or median) values.

### Statistical models

Generalised linear mixed models were used to estimate the ICCs with cluster (general practice) modelled as the random effect. Both adjusted and unadjusted ICCs were estimated, with adjustments made for age, sex, location, and deprivation quintiles. All clinical measures were presented in both continuous and dichotomised form.

For continuous outcomes, a mixed-effects linear model was fitted and the ICC was estimated as the ratio of the between-cluster variance (of the outcome) to the total variance of the outcome.

For binary outcomes, a mixed-effects linear model was fitted to estimate the natural ICC on the proportion scale, whilst a mixed-effects logistic regression was fitted to estimate the latent ICC.

To estimate the WPC, IPC, and CA, a generalised linear mixed model was used, with two random effects – one for cluster (general practice) and one for a cluster by period interaction.

All analysis was performed using Stata 13 (StataCorp, College Station, TX, USA). Linear models were fitted using the mixed command, and logistic models fitted using the melogit command. Estimates of the ICC, WPC, and IPC were produced using the estat function.

### Search of previous CRCTs

A systematic search of previous CRCTs investigating diabetes in primary care in the UK was carried out in order to compare the results from this analysis to values used in previous CRCTs.

The following sources were used: Medline (1950 to week 2 of May 2013), Medline InProcess (May 2013), and Google Scholar (May 2013). The searches were conducted in May 2013. The following phrases were used: type-II diabetes, type-2 diabetes, diabetes mellitus, diabetes mellitus non-insulin-dependent, adult-onset diabetes mellitus, cluster trial, clustered trial, cluster analysis, cluster analyses, clustering, disease clustering, cluster RCT, and cluster randomised (randomized) controlled trial. The search was limited to the English language.

Studies from all fields of research were included if they described a CRCT that had taken place, or was planned to take place, that used UK general practices as the unit of randomisation. Studies were included if at least one of the trial outcomes were: HbA1c levels, systolic blood pressure, diastolic blood pressure, BMI, total cholesterol, HDL cholesterol, the prescription of insulin, or the onset of microvascular and macrovascular outcomes.

Since the focus is on the ICCs used in the design of a CRCT, all trials in which individuals were the unit of randomisation were excluded from the study. All trials that did not take place in the UK were also excluded since ICC estimates may be affected by the country in which the trial is taking place. All trials with unspecified outcomes were excluded. Trials that aim to prevent the onset of diabetes were also excluded. Any duplicate or follow-on publications from the same trial were included as a single study.

Titles and abstracts retrieved from the search process were screened to obtain relevant trials. Full articles were then read and classified as either included or excluded. All included articles were then used for data extraction. The extracted information consisted of: study authors, outcome used, value of ICC used in the sample size calculation, standard deviation used in the sample size calculation (where appropriate), and the ICC estimated from the trial data (if reported).

## Results

### Analysis of THIN data

A summary of patient and practice characteristics is given in Table 1. A total of 112,633 patients from 430 practices covering all areas of the UK, were included in the study. The socioeconomic status was fairly balanced across the categories. The median value of HbA1c (%) (7.05) was lower than the mean value (7.35), highlighting the positive skewness that is exhibited by the variable. Atrial fibrillation was the most common clinical outcome (1.06 %), whilst chronic kidney disease was the least common (0.35 %).

**Table 1:** Summary of study population (THIN database) by practice and patient-level characteristics

Practice characteristics	
Number of general practices	430
General practice size^a^, median [IQR]	241 [150–351]
Location, *N* (%)	
England	322 (75)
Northern Ireland	21 (5)
Scotland	56 (13)
Wales	31 (7)
Patient characteristics	
Number of patients	112,633
Age, median [IQR]	70 [60–78]
Sex (male), *N* (%)	61,944 (55)
Location, *N* (%)	
England	88,838 (79)
Northern Ireland	3464 (3)
Scotland	12,461 (11)
Wales	7870 (7)
Deprivation quintiles, *N* (%)	
1 (most affluent)	23,853 (21)
2	23,106 (21)
3	23,031 (20)
4	22,054 (20)
5 (most deprived)	16,352 (15)
Unknown	4237 (4)
Clinical measures	
HbA1c (%), median [IQR]	7.05 [6.4–7.9]
HbA1c (%), mean (SD)	7.35 (1.41)
Systolic blood pressure (mmHg), mean (SD)	134 (16)
Diastolic blood pressure (mmHg), mean (SD)	75 (10)
BMI (kg/m^2^), median [IQR]	29.8 [26.4–34.1]
Total cholesterol (mmol/L), median [IQR]	4.1 [3.5–4.7]
HDL cholesterol (mmol/L), median [IQR]	1.19 [1.00–1.40]
Medication, *N* (%)	
Insulin	66,530 (59.1)
Other hypoglycaemic medication	33,061 (29.4)
Clinical outcomes, *N* (%)	
Atrial fibrillation	1101 (1.058)
Chronic kidney disease	381 (0.349)
Chronic obstructive pulmonary disease	846 (0.801)
Ischaemic heart disease	967 (1.109)
Peripheral vascular disease	575 (0.545)
Stroke	458 (0.426)

*BMI* body mass index, *HbA1c* glycosylated haemoglobin, *HDL* high-density lipoprotein, *IQR* interquartile range, *SD* standard deviation. Note: the percentage corresponds to the number of applicable patients, and so the total may not be identical for each outcome variable. ^a^Here general practice size corresponds to the number of patients within the general practice who have satisfied the entry criteria (patients who had C10F Read (version 2) code for diabetes entered on the Vision GP patient management system or other codes specifying type-2 diabetes)

Table 2 summarises the proportion of patients whose clinical measures exceed the dichotomised value of the outcomes. Of the participants with a recording for HbA1c, over one third (34.2 %) had an HbA1c % exceeding 7.5 %. It was also found that over one half (57.2 %) exceeded the target systolic blood pressure of 130 mmHg whilst approximately one quarter (25.2 %) exceeded 140 mmHg. A large proportion (83.1 %) of the population were categorised as being overweight (>25 kg/m^2^) (34.8 %), obese (>30 kg/m^2^) (27.3 %), or morbidly obese (>35 kg/m^2^) (21.0 %).

**Table 2:** Summary statistics for clinical measures of included patients from THIN database in binary form

Outcome	Number of observations	Number of patients exceeding measurement
Clinical measures, *N* (%)		
HbA1c (%) (>7.5)	101,412	34,723 (34.2)
Systolic blood pressure (mmHg) (>140)	86,918	21,865 (25.2)
Systolic blood pressure (mmHg) (>130)	86,918	49,697 (57.2)
Diastolic blood pressure (mmHg) (>80)	86,918	20,403 (23.5)
BMI (kg/m^2^) (>30)	86,681	41,880 (48.3)
BMI (kg/m^2^) (>25)	86,681	72,062 (83.1)
Total cholesterol (mmol/L) (>4)	92,089	46,378 (50.4)
HDL cholesterol (mmol/L) (<1.2)	80,690	40,653 (50.3)

*BMI* body mass index *HbA1c* glycosylated haemoglobin, *HDL* high-density lipoprotein

**Fig. 1:**
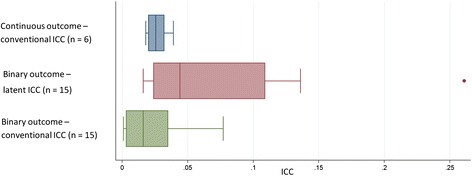
Box plot highlighting the median, interquartile range, and range of the intra-cluster correlation coefficients (ICCs) that were estimated for continuous and binary clinical outcomes from both linear and logistic models (*n* = number of outcomes that had estimate of the ICC)

The variation of both the clinical outcomes and clinical measures across practices is given in Table 3. The interquartile range represents the practice mean outcome for the central 50 % of practices. ICC estimates and corresponding standard errors (SE) for clinical measures of continuous nature are given in Table 4 and compared further in Fig. 1. For clinical measurements, in continuous form, the ICCs had a median of 0.026 [IQR 0.020–0.032] and were similar when adjusting for confounding factors (median 0.025, IQR 0.020–0.029). The ICC for HbA1c was estimated to be 0.032 (SE 0.003) when using an unadjusted model and 0.032 (SE 0.003) after adjustment for patient-level factors.

**Table 3:** Summary of the variation of practice average values from included patients from THIN database

Outcomes, median [IQR]	Practice average
Age	68.4 [66.9–69.8]
Sex, (%)	Male, 55 [53–58]
Clinical measures	
HbA1c (%), median	7.05 [6.9–7.2]
Systolic blood pressure (mmHg), median	135 [132–137.5]
Diastolic blood pressure (mmHg), median	76 [74–78]
BMI (kg/m^2^), median	29.9 [29.2–30.6]
Total cholesterol (mmol/L), median	4.10 [3.90–4.18]
HDL cholesterol (mmol/L), median	1.18 [1.10–1.20]
Medication, (%)	
Prescribed insulin	57.8 [48.0–68.6]
Prescribed other hypoglycaemic medication	29.2 [21.8–36.7]
Clinical outcomes (%)	
Atrial fibrillation	0.909 [0.476–1.497]
Chronic kidney disease	0.000 [0.000–0.560]
Chronic obstructive pulmonary disease	0.708 [0.000–1.205]
Ischaemic heart disease	0.389 [0.000–0.862]
Peripheral vascular disease	0.371 [0.000–0.800]
Stroke	0.000 [0.000–0.667]

*BMI* body mass index, *HbA1c* glycosylated haemoglobin, *HDL* high-density lipoprotein, *IQR* interquartile range. Note: the average outcome value/clinical measure was calculated for each practice using mean or median as appropriate. The average practice values were then summarised across practices by the median and interquartile range

**Table 4:** Intra-cluster correlation coefficients (ICCs) for continuous outcomes for included patients from THIN database

Outcome	ICC from unadjusted model (SE)	ICC from adjusted model (SE)
Clinical measures		
HbA1c (%)	0.032 (0.003)	0.032 (0.003)
Systolic blood pressure (mmHg)	0.031 (0.003)	0.029 (0.002)
Diastolic blood pressure (mmHg)	0.039 (0.003)	0.039 (0.003)
BMI (kg/m^2^)	0.020 (0.002)	0.022 (0.002)
Total cholesterol (mmol/L)	0.020 (0.002)	0.018 (0.002)
HDL cholesterol (mmol/L)	0.021 (0.002)	0.020 (0.002)

*BMI* body mass index, *HbA1c* glycosylated haemoglobin, *HDL* high-density lipoprotein, *SE* standard error

After dichotomising, the ICCs of clinical measures had a median latent ICC of 0.037 [IQR 0.023–0.055] and a median natural ICC on the proportion scale of 0.028 [IQR 0.018–0.039]. Clinical outcomes had a median latent ICC of 0.094 [IQR 0.027–0.136] and a median natural ICC on the proportion scale of 0.003 [IQR 0.001–0.005]. When comparing two clinical outcomes with similar prevalence, it is expected that the outcome with a larger IQR of the practice average would have a larger ICC. This is consistent with the larger natural and latent ICCs (Table 5) that are associated with COPD compared to IHD, both of which have a prevalence of around 1 % (Table 1). Figure 1 further highlights that latent ICCs were larger than natural ICCs on the proportion scale for binary outcomes, but also that the range of latent ICCs is higher than natural ICCs.

**Table 5:** Intra-cluster correlation coefficients (ICCs) for binary outcomes for included patients from THIN database

		Logistic regression model		Linear model	
Outcome	Prevalence of outcome	ICC from unadjusted model (SE)	ICC from adjusted model (SE)	ICC from unadjusted model (SE)	ICC from adjusted model (SE)
Clinical measures					
HbA1c (%) (>7.5)	0.342	0.035 (0.003)	0.037 (0.003)	0.026 (0.002)	0.027 (0.002)
Systolic blood pressure (mmHg) (>140)	0.252	0.069 (0.005)	0.066 (0.005)	0.039 (0.003)	0.036 (0.003)
Systolic blood pressure (mmHg) (>130)	0.572	0.047 (0.004)	0.044 (0.003)	0.038 (0.003)	0.035 (0.003)
Diastolic blood pressure (mmHg) (>80)	0.235	0.086 (0.006)	0.088 (0.007)	0.046 (0.004)	0.045 (0.003)
BMI (kg/m^2^) (>30)	0.483	0.019 (0.002)	0.022 (0.002	0.015 (0.001)	0.016 (0.002)
BMI (kg/m^2^) (>25)	0.831	0.022 (0.002)	0.022 (0.002)	0.011 (0.001)	0.010 (0.001)
Total cholesterol (mmol/L) (>4)	0.504	0.025 (0.002)	0.024 (0.002)	0.020 (0.002)	0.019 (0.002)
HDL cholesterol (mmol/L) (<1.2)	0.503	0.035 (0.002)	0.036 (0.003)	0.029 (0.002)	0.027 (0.002)
Medication					
Taking of insulin	0.591	0.113 (0.007)	0.109 (0.007)	0.081 (0.006)	0.077 (0.005)
Clinical outcomes					
Atrial fibrillation	0.01058	0.020 (0.009)	0.016 (0.009)	0.001 (0.000)	0.001 (0.000)
Chronic kidney disease	0.00349	0.140 (0.026)	0.136 (0.026)	0.003 (0.000)	0.003 (0.000)
Chronic obstructive pulmonary disease	0.00801	0.070 (0.015)	0.064 (0.015)	0.002 (0.000)	0.002 (0.000)
Ischaemic heart disease	0.01109	0.027 (0.011)	0.027 (0.011)	0.001 (0.000)	0.001 (0.000)
Peripheral vascular disease	0.00545	0.130 (0.020)	0.124 (0.020)	0.005 (0.001)	0.005 (0.001)
Stroke	0.00426	0.274 (0.031)	0.261 (0.031)	0.010 (0.001)	0.010 (0.001)

*BMI* body mass index, *HbA1c* glycosylated haemoglobin, *HDL* high-density lipoprotein, *SE* standard error

Estimates of the WPC, IPC, and CA for the two-period study design are given in Table 6. For HbA1c, the correlation between two patients during the same (12-month) time period (WPC) was estimated at 0.035 (SE 0.003). The correlation between two patients at different (12-month) time periods (IPC) is 0.019 (SE 0.003). There is evidence to suggest that the variance component related to time period is non-zero, and so the correlation of observations seems to decay over time. Excluding HbA1c, in the two-period (each of 15 months) design, the decay of correlation is further highlighted by the median WPC (0.021, IQR 0.021–0.032) and median IPC (0.018, IQR 0.013–0.021).

**Table 6:** Estimates of the within-period and inter-period correlation for included patients from THIN database from two consecutive periods

	Unadjusted model			Adjusted model		
Outcome	WPC	IPC	CA	WPC	IPC	CA
	(SE)	(SE)		(SE)	(SE)	
HbA1c^a^ (%)	0.035	0.019	0.546	0.035	0.019	0.539
	(0.003)	(0.003)		(0.003)	(0.003)	
Systolic blood pressure^b^ (mmHg)	0.032	0.021	0.649	0.030	0.019	0.632
	(0.003)	(0.003)		(0.002)	(0.003)	
Diastolic blood pressure^b^ (mmHg)	0.040	0.028	0.692	0.040	0.026	0.662
	(0.003)	(0.004)		(0.003)	(0.004)	
BMI^b^ (kg/m^2^)	0.021	0.013	0.612	0.022	0.016	0.747
	(0.002)	(0.002)		(0.002)	(0.002)	
Total cholesterol^b^ (mmol/L)	0.021	0.010	0.486	0.021	0.006	0.281
	(0.002)	(0.002)		(0.002)	(0.002)	
HDL cholesterol^b^ (mmol/L)	0.021	0.018	0.876	0.020	0.017	0.889
	(0.002)	(0.002)		(0.002)	(0.002)	

*BMI* body mass index, *CA* cluster autocorrelation, *HbA1c* glycosylated haemoglobin, *HDL* high-density lipoprotein, *IPC* inter-period correlation, *SE* standard error, *WPC* within-period correlation. ^a^Two consecutive 12-month periods were used. ^b^Two consecutive 15-month periods were used

The median cluster autocorrelation (excluding HbA1c) is 0.649 [IQR 0.612–0.692], with total cholesterol having the smallest value – indicating that correlation of total cholesterol observations for patients in different time periods is much smaller than the correlation of observations in the same time period. Adjusting for covariates had some impact on correlation estimates. For total cholesterol, the CA in the adjusted model (0.281) was much lower than the unadjusted model (0.486). Conversely, HbA1c had much higher CA in the adjusted model (0.747) than in the unadjusted model (0.612).

### Systematic search

Our search strategy found 133 relevant articles. From this, 70 articles were of irrelevant outcome or trial type (individually randomised design, genetics of diabetes, cross-sectional studies, etc.), 36 were excluded due to the population of the trials (not of UK origin), 7 articles were screening programmes, 6 aimed to prevent diabetes, and 2 articles were excluded as they measure prevalence of diabetes. Of the 12 trials remaining, 3 duplicates were removed, leaving 9 articles that met the inclusion criteria (see Additional file 1).

One CRCT used the cluster as unit of randomisation but did not use an ICC when calculating sample size [46]. Of the remaining eight CRCTs, two CRCTs [39, 47] used multiple outcomes and calculated sample sizes for each outcome of relevance. Seven CRCTs [14, 39, 47–51] used HbA1c as an outcome measure, three [38, 39, 47] used systolic blood pressure, and two [39, 47] used cholesterol. However, cholesterol was not used as a sole outcome measure, only as secondary measure alongside both HbA1c and blood pressure. Of these eight CRCTs, two [38, 39] used a binary outcome, and seven [14, 39, 47–51] used a continuous outcome (one used both a binary and continuous outcome [39]).

The median [IQR] ICC used to power the study for trials in which HbA1c % was the primary outcome was 0.047 [0.047–0.05] (Table 7). The two CRCTs [39, 47] in which total cholesterol (mmol/L) was the main outcome used 0.047 and 0.06 (binary outcome) as the ICC whilst the three CRCTs using blood pressure (mmHg) as the main outcome [38, 39, 47] used ICCs of 0.001 (binary outcome), 0.02 (binary outcome), and 0.035. The standard deviation of HbA1c % used was reported in six trials [14, 39, 47, 49–51], of which the mean value was 1.7. The results of this paper found a similar standard deviation of 1.4 for HbA1c %, whereas the ICC found by this paper was lower (0.032 versus 0.047).

**Table 7:** Summary of systematic search of intra-cluster correlation coefficients (ICCs) used in previous trials

Trial author	Outcome used	ICC used	Standard deviation used	ICC reported
Bellary et al. [47]	HbA1c	0.05	2.1	
Dallosso et al. [50]	HbA1c	0.05	1.5	
Khunti et al. [14]	HbA1c	0.05	2.0	0.02 (0.00–0.08)
Mathers et al. [49]	HbA1c	0.047	1.5	
Smith et al. [39]	HbA1c	0.001	1.6	
Smith et al. [51]	HbA1c	0.047	1.5	
Sturt et al. [48]	HbA1c	0.047		0.0253
Bebb et al. [38]	BP	0.02	Binary outcome	0.035
Bellary et al. [47]	SBP	0.035	21.25	
Smith et al. [39]	SBP	0.001	Binary outcome	
Bellary et al. [47]	Cholesterol	0.05	1.1	
Smith et al. [39]	Cholesterol	0.06	Binary outcome	

Here glycosylated haemoglobin (HbA1c) represents a difference in average HbA1c levels between patients in the control and intervention groups. SBP represents systolic blood pressure, BP represents blood pressure – where a reading of both systolic and diastolic blood pressure was taken, and cholesterol represents total cholesterol level

Only three trials reported ICCs from their analysis [14, 38, 48]. Two trials reported ICCs for HbA1c % [14, 48], with ICCs of 0.0253 and 0.02 (95 % CI 0.00–0.08), and one trial [38] reported an ICC for blood pressure of 0.035. For the two trials that reported the ICC, the reported value was lower than the value used in the initial sample size calculation, whilst for blood pressure the reported value was notably higher. However, for the trial that estimated an ICC for blood pressure [38], it was not clear what method was used to estimate this value.

## Discussion

Using THIN database, we have estimated ICCs for a variety of outcomes associated with type-2 diabetes. We are the first to report time-dependent correlations, the IPC and WPC, which can be used in the design of cluster cross-over and stepped wedge CRCTs. For binary outcomes, we reported both the latent ICC (an ICC from a logistic model) and the natural ICC on the proportion scale (an ICC from a linear model).

These results are primarily applicable for planned CRCTs aimed at the general practice level in the UK, but in the absence of other estimates, may be useful more widely. We found that the ICC for HbA1c used in the design of trials tended to be larger than that estimated here.

### Intra-cluster correlation coefficients

ICCs were calculated for continuous and dichotomous clinical measurements and outcomes, using both adjusted and unadjusted models. This includes ICCs for continuous outcomes and ICCs for binary outcomes. Upon adjusting for age, sex, location, and deprivation quintiles, the ICCs were generally similar to the ICCs estimated from the unadjusted models (HbA1c 0.032 versus 0.032). Adjusting for confounding factors also had minimal impact on the standard error of the ICCs (HbA1c 0.003 versus 0.003).

There was a noticeable difference between natural ICCs and latent ICCs for binary outcomes. Latent ICCs estimated for clinical events were much larger than their corresponding natural ICC. Similar results were found by Wu et al. [52], who found that ICCs were smaller when modelled using linear regression than logistic regression.

For binary outcomes it is important to note that natural ICCs (an ICC from a linear model) are smaller for cases in which the prevalence’s are low [35, 53]. Here all clinical outcomes chosen were rare events and consequently had small prevalence’s. Since the dichotomised values were chosen to reflect typical values in relation to type-2 diabetes, the prevalence’s of these were naturally larger – resulting in a larger ICC.

Due to the importance of the prevalence on the natural ICC, care should be taken to ensure that an appropriate ICC is used. If the prevalence in a planned trial differs greatly from the prevalence used here, sample size calculations using the natural ICC from these results may be inaccurate

Since latent ICCs for dichotomous outcomes, are estimated using logistic regression, they are on a log-odds scale and so are defined on a different scale to a natural ICC [35, 52]. A latent ICC estimated in this manner will refer to an unobservable latent scale, rather than the correlation of observations within a cluster, and so would not be a relevant ICC for use in the design stage of a trial. Eldridge et al. [21] provide a table that allows some ICCs on this logistic scale to be converted into a natural ICC for a selection of prevalence’s.

### Previous trials

Many authors discuss the most appropriate methods and models that should be used to model ICCs in situations in which the outcome is binary [35, 52, 54], and there are numerous cases in which previous authors have correctly estimated ICCs for binary outcomes using linear models for future trialists to use [34, 55–57]. However, there are still some situations where a logistic model is used [58–60]. The differences between the natural ICC and the latent ICC are also considered by Merlo et al. [61] who note that since the natural ICC depends on the prevalence of the outcome; any comparisons made regarding the magnitude of clustering should be made using the latent ICC. We agree that that care should be taken when using the natural ICC to describe the extent of clustering in a trial with binary outcomes; however, we cannot recommend that the latent ICC is used directly in the design of future trials.

The number of previous cluster trials involving type-2 diabetes that have reported ICCs from their results is rather small, which will leave future trialists using ad hoc values or conservative values. The ICCs found in this paper were smaller than that often used in trials, but more consistent with the ICCs that were reported from the results of previous trials. The ICC for HbA1c %, the most common outcome in a trial involving type-2 diabetes, was found to be 0.032 (SD 0.003). Trials in which the primary outcome is binary should use an ICC from a linear model when estimating a required sample size, and not one obtained from a logistic model, even if the data will be analysed using a logistic model.

### Inter-period correlation coefficients

It is emerging that cluster designs require not only estimates of within-cluster correlation measures, but some value of how this correlation decays over time [29, 62]. We have attempted in part to address this issue and are the first to provide estimates of the inter-period correlation and the within-period correlation alongside ICCs. However, we have only provided these estimates for continuous outcomes and we have only provided estimates assuming a cross-sectional study design. Clearly, many studies use a cohort design and many studies contain a primary outcome that is dichotomous in nature. However, estimation of correlation coefficients for binary outcomes are more complex due to the change of scale; and adding a cohort structure would increase complexity, as it would also be necessary to allow for within-person correlation.

The IPC and WPC may also be reported as the CA. It has been established that the sample size is directly impacted by the CA [37]. No guidelines exist for reasonable values of the CA, but values of 0.8 and 1.0 have previously been used [28, 63]. Here we have shown that for our study design, the CA may be smaller than these estimates.

Ignoring the IPC and CA in sample size calculations may lead to incorrect estimates of the required number of clusters in a CRT [29] or to underpowered studies [28]. Studies in which the IPC differs to the WPC should ensure that the estimates of *ρ* for use in Eq. 1 stem from the WCC estimated via Eq. 3, and not from an ICC estimated by Eq. 2.

### Future research

It has been established that the ICC, IPC, and CA are necessary for sample size calculations for CRCTs. However, there is opportunity for future research into the IPC and the impact of time between observations in the model for CRCTs. It is perhaps naïve to assume a fixed correlation between observations in a cluster trial regardless of the time between these. Instead, this correlation should depend on time, and this length of time may be important. It is not known what impact changing the length of time period or the length of the study period would have on the IPC. Additionally, the IPC used to direct a sample size calculation should be calculated from a dataset using a similar time period and study length. The motivating idea behind additional correlation types is repeated cross-sectional designs such as the cluster cross-over design and the stepped wedge design. However, these results may indicate that sample size in parallel CRCTs should also acknowledge that correlation may be time-dependent. Future research is likely to show that recognising the decay in correlation over time in the model would increase power in parallel designs.

### Limitations

There are limitations that may arise from using routine data from general practices. It is not always possible to distinguish between follow-up care for a first clinical event (e.g. myocardial infarction) from a second event as they may have been coded in an identical manner. This means that patients who had suffered an event prior to the study inclusion period would have to be excluded from the analysis. There is also the possibility of misclassification as type-2 diabetes rather than type-1 diabetes due to coding errors, which could lead to younger patients being included in the study unintentionally.

Since THIN dataset consists of data from general practices only, the results can only be adjusted for variables that are recorded by the practice. The quality of service may vary between practices and so there may be situations in which clinical measures are monitored in different intervals which, along with quality of reporting and recording of measurements, could lead to an inconsistency.

Although the reporting of clinical measures during the 15-month cross-section that was chosen as the inclusion period was high, the length of the cross-section may not accurately represent the length of trials in practice.

## Conclusions

An estimate of the ICC is vital when calculating the sample size requirement in a pretrial calculation [21]. We estimated ICCs for a range of clinical outcomes related to type-2 diabetes that would be useful for planning a trial in UK primary care. The primary outcome used in type-2 diabetes trials is often HbA1c, for which we estimated an ICC of 0.032. We have also illustrated how the methodology described here could be extended for other outcomes or disease settings.

For binary outcomes, the results show careful consideration is needed when estimating the ICC. This is because, in a trial with a dichotomous outcome, the ICC used at the design stage should refer to the variation in the observed data rather than the underlying logistic scale. Despite the analysis of binary outcomes being usually conducted via a logistic regression model, the latent ICC obtained from such model should not be used for sample size calculations. Rather, the ICC used in the design stage of a trial should be estimated from a linear mixed model on the natural scale.

In cluster trials with repeated cross-sections, observations are taken over multiple time periods. It is likely that observations within a cluster within the same time period are more highly correlated than observations from different time periods. The inter-period correlation and within-period correlation provides an estimate of how this correlation deteriorates over time. We are the first to report estimates of the IPC and WPC and we have illustrated how these differ from the ICC. It may be important to acknowledge the degeneration of correlation over time in repeated cross-sectional studies.

## Additional file

Additional file 1: Flow diagram of included trials for systematic search of trials undertaken in primary care in type-2 diabetes. (PNG 17 kb)

## Abbreviations

BMI: Body Mass Index
CA: Cluster Autocorrelation
CRCT: Clustered Randomised Controlled Trial
ICC: Intra-cluster Correlation Coefficient
IPC: Inter-period Correlation
RCT: Randomised Controlled Trial
THIN: The Health Improvement Network
WCC: Within-cluster Correlation
WPC: Within-period Correlation

## References

[1] Danaei G, Finucane MM, Lu Y, Singh GM, Cowan MJ, Paciorek CJ (2011). National, regional, and global trends in fasting plasma glucose and diabetes prevalence since 1980: systematic analysis of health examination surveys and epidemiological studies with 370 country-years and 2.7 million participants. Lancet.

[2] Donner A, Kong AP (2000). Design and analysis of cluster randomization trials in health research.

[3] Edwards SJ, Braunholtz DA, Lilford RJ, Stevens AJ (1999). Ethical issues in the design and conduct of cluster randomised controlled trials. BMJ.

[4] Donner A (1998). Some aspects of the design and analysis of cluster randomization trials. J R Stat Soc: Ser C: Appl Stat.

[5] Campbell MJ, Walters SJ (2014). How to design, analyse and report cluster randomised trials in medicine and health related research.

[6] Puffer S, Torgerson DJ, Watson J (2005). Cluster randomized controlled trials. J Eval Clin Pract.

[7] Eldridge S, Kerry S. A practical guide to cluster randomised trials in health services research. John Wiley & Sons Ltd, Wiley; 2012.

[8] Lancaster GA, Campbell MJ, Eldridge S, Farrin A, Marchant M, Muller S (2010). Trials in primary care: statistical issues in the design, conduct and evaluation of complex interventions. Stat Methods Med Res.

[9] Campbell MJ (2000). Cluster randomized trials in general (family) practice research. Stat Methods Med Res.

[10] Donner A, Klar N (1996). Statistical considerations in the design and analysis of community intervention trials. J Clin Epidemiol.

[11] Kerry SM, Bland JM (1998). Trials which randomize practices II: sample size. Fam Pract.

[12] Hayes RJ, Bennett S (1999). Simple sample size calculation for cluster-randomized trials. Int J Epidemiol.

[13] Hayes R, Moulton L (2009). Cluster randomised trials.

[14] Khunti K, Gray LJ, Skinner T, Carey ME, Realf K, Dallosso H (2012). Effectiveness of a diabetes education and self management programme (DESMOND) for people with newly diagnosed type 2 diabetes mellitus: three year follow-up of a cluster randomised controlled trial in primary care. BMJ.

[15] Foster GD, Linder B, Baranowski T, Cooper DM, Goldberg L, Harrell JS (2010). A school-based intervention for diabetes risk reduction. N Engl J Med.

[16] Shahbazian H, Latifi SM, Jalali MT, Shahbazian H, Amani R, Nikhoo A (2013). Metabolic syndrome and its correlated factors in an urban population in South West of Iran. J Diabetes Metab Disord.

[17] Heisler M, Hofer TP, Schmittdiel JA, Selby JV, Klamerus ML, Bosworth HB (2012). Improving blood pressure control through a clinical pharmacist outreach program in patients with diabetes mellitus in 2 high-performing health systems: the adherence and intensification of medications cluster randomized, controlled pragmatic trial. Circulation.

[18] Echouffo-Tcheugui J, Simmons R, Williams K, Barling R, Prevost AT, Kinmonth A (2009). The ADDITION-Cambridge trial protocol: a cluster-randomised controlled trial of screening for type 2 diabetes and intensive treatment for screen-detected patients. BMC Public Health.

[19] Hansen LJ, Siersma V, Beck-Nielsen H, de Fine Olivarius N (2013). Structured personal care of type 2 diabetes: a 19 year follow-up of the study Diabetes Care in General Practice (DCGP). Diabetologia.

[20] Hemming K, Girling AJ, Sitch AJ, Marsh J, Lilford RJ (2011). Sample size calculations for cluster randomised controlled trials with a fixed number of clusters. BMC Med Res Methodol.

[21] Eldridge SM, Ukoumunne OC, Carlin JB (2009). The intra-cluster correlation coefficient in cluster randomized trials: a review of definitions. Int Stat Rev.

[22] Campbell MK, Mollison J, Steen N, Grimshaw JM, Eccles M (2000). Analysis of cluster randomized trials in primary care: a practical approach. Fam Pract.

[23] Bell ML, McKenzie JE (2013). Designing psycho-oncology randomised trials and cluster randomised trials: variance components and intra-cluster correlation of commonly used psychosocial measures. Psycho-Oncology.

[24] Parienti JJ, Kuss O (2007). Cluster-crossover design: a method for limiting clusters level effect in community-intervention studies. Contemp Clin Trials.

[25] Hemming K, Haines TP, Chilton PJ, Girling AJ, Lilford RJ (2015). The stepped wedge cluster randomised trial: rationale, design, analysis, and reporting. BMJ.

[26] Martin J, Taljaard M, Girling A, Hemming K (2016). Systematic review finds major deficiencies in sample size methodology and reporting for stepped-wedge cluster randomised trials. BMJ Open.

[27] Hooper R, Bourke L (2014). The dog-leg: an alternative to a cross-over design for pragmatic clinical trials in relatively stable populations. Int J Epidemiol.

[28] Hooper R, Bourke L (2015). Cluster randomised trials with repeated cross sections: alternatives to parallel group designs. BMJ.

[29] Taljaard M, Teerenstra S, Ivers NM, Fergusson DA. Substantial risks associated with few clusters in cluster randomized and stepped wedge designs. Clin Trials (London, England). 2016. doi:10.1177/1740774516634316. 10.1177/1740774516634316 26940696

[30] Girling AJ, Hemming K (2016). Statistical efficiency and optimal design for stepped cluster studies under linear mixed effects models. Stat Med.

[31] Ukoumunne OC, Thompson SG (2001). Analysis of cluster randomized trials with repeated cross-sectional binary measurements. Stat Med.

[32] Turner RM, White IR, Croudace T (2007). Analysis of cluster randomized cross-over trial data: a comparison of methods. Stat Med.

[33] Webb DR, Khunti K, Gray LJ, Srinivasan BT, Farooqi A, Wareham N (2012). Intensive multifactorial intervention improves modelled coronary heart disease risk in screen-detected Type 2 diabetes mellitus: a cluster randomized controlled trial. Diabet Med.

[34] Taljaard M, Donner A, Villar J, Wojdyla D, Velazco A, Bataglia V (2008). Intracluster correlation coefficients from the 2005 WHO Global Survey on Maternal and Perinatal Health: implications for implementation research. Paediatr Perinat Epidemiol.

[35] Gulliford MC, Adams G, Ukoumunne OC, Latinovic R, Chinn S, Campbell MJ (2005). Intraclass correlation coefficient and outcome prevalence are associated in clustered binary data. J Clin Epidemiol.

[36] Research CM (2012). Our data.

[37] Teerenstra S, Eldridge S, Graff M, de Hoop E, Borm GF (2012). A simple sample size formula for analysis of covariance in cluster randomized trials. Stat Med.

[38] Bebb C, Kendrick D, Coupland C, Madeley R, Stewart J, Brown K (2007). A cluster randomised controlled trial of the effect of a treatment algorithm for hypertension in patients with type 2 diabetes. Br J Gen Pract.

[39] Smith SM, Paul G, Kelly A, Whitford DL, O’Shea E, O’Dowd T (2011). Peer support for patients with type 2 diabetes: cluster randomised controlled trial. BMJ.

[40] (NICE) NIfHaCE. Type 2 diabetes: the management of type 2 diabetes. NICE guidelines [CG87]; John Wiley & Sons Ltd; 2009.

[41] Currie CJ, Peters JR, Tynan A, Evans M, Heine RJ, Bracco OL, et al. Survival as a function of HbA1c in people with type 2 diabetes: a retrospective cohort study. Lancet. 375(9713);481–9. http://dx.doi.org/10.1016/S0140-6736(09)61969-3. 10.1016/S0140-6736(09)61969-3 20110121

[42] Kirby M (2009). Achieving effective lipid management in diabetes. Br J Prim Care Nurs.

[43] Association AD (2007). Standards of medical care in diabetes—2007. Diabetes Care.

[44] (NICE) NIfHaCE. Quality and outcomes framework indicators. 2004. https://www.nice.org.uk/Standards-and-Indicators/QOFIndicators. Accessed 11 June 2015.

[45] Maguire A, Blak BT, Thompson M (2009). The importance of defining periods of complete mortality reporting for research using automated data from primary care. Pharmacoepidemiol Drug Saf.

[46] O’Hare JP, Raymond NT, Mughal S, Dodd L, Hanif W, Ahmad Y (2004). Evaluation of delivery of enhanced diabetes care to patients of South Asian ethnicity: the United Kingdom Asian Diabetes Study (UKADS). Diabet Med.

[47] Bellary S, O’Hare JP, Raymond NT, Gumber A, Mughal S, Szczepura A (2008). Enhanced diabetes care to patients of south Asian ethnic origin (the United Kingdom Asian Diabetes Study): a cluster randomised controlled trial. Lancet.

[48] Sturt JA, Whitlock S, Fox C, Hearnshaw H, Farmer AJ, Wakelin M (2008). Effects of the Diabetes Manual 1:1 structured education in primary care. Diabet Med.

[49] Mathers N, Ng CJ, Campbell MJ, Colwell B, Brown I, Bradley A. Clinical effectiveness of a patient decision aid to improve decision quality and glycaemic control in people with diabetes making treatment choices: a cluster randomised controlled trial (PANDAs) in general practice. BMJ Open. 2012;2(6). doi: 10.1136/bmjopen-2012-001469. 10.1136/bmjopen-2012-001469 PMC3532975 23129571

[50] Dallosso HM, Eborall HC, Daly H, Martin-Stacey L, Speight J, Realf K (2012). Does self monitoring of blood glucose as opposed to urinalysis provide additional benefit in patients newly diagnosed with type 2 diabetes receiving structured education? The DESMOND SMBG randomised controlled trial protocol. BMC Fam Pract.

[51] Smith S, Bury G, O’Leary M, Shannon W, Tynan A, Staines A (2004). The North Dublin randomized controlled trial of structured diabetes shared care. Fam Pract.

[52] Wu S, Crespi CM, Wong WK (2012). Comparison of methods for estimating the intraclass correlation coefficient for binary responses in cancer prevention cluster randomized trials. Contemp Clin Trials.

[53] Pagel C, Prost A, Lewycka S, Das S, Colbourn T, Mahapatra R (2011). Intracluster correlation coefficients and coefficients of variation for perinatal outcomes from five cluster-randomised controlled trials in low and middle-income countries: results and methodological implications. Trials.

[54] Yelland LN, Salter AB, Ryan P, Laurence CO (2011). Adjusted intraclass correlation coefficients for binary data: methods and estimates from a cluster-randomized trial in primary care. Clin Trials (London, England).

[55] Adams G, Gulliford MC, Ukoumunne OC, Eldridge S, Chinn S, Campbell MJ (2004). Patterns of intra-cluster correlation from primary care research to inform study design and analysis. J Clin Epidemiol.

[56] Roudsari B, Fowler R, Nathens A (2007). Intracluster correlation coefficient in multicenter childhood trauma studies. Inj Prev.

[57] Thompson DM, Fernald DH, Mold JW (2012). Intraclass correlation coefficients typical of cluster-randomized studies: estimates from the Robert Wood Johnson Prescription for Health projects. Ann Fam Med.

[58] Kul S, Vanhaecht K, Panella M (2014). Intraclass correlation coefficients for cluster randomized trials in care pathways and usual care: hospital treatment for heart failure. BMC Health Serv Res.

[59] Moineddin R, Matheson FI, Glazier RH (2007). A simulation study of sample size for multilevel logistic regression models. BMC Med Res Methodol.

[60] Turner RM, Omar RZ, Thompson SG (2001). Bayesian methods of analysis for cluster randomized trials with binary outcome data. Stat Med.

[61] Merlo J, Chaix B, Ohlsson H, Beckman A, Johnell K, Hjerpe P (2006). A brief conceptual tutorial of multilevel analysis in social epidemiology: using measures of clustering in multilevel logistic regression to investigate contextual phenomena. J Epidemiol Community Health.

[62] Giraudeau B, Ravaud P, Donner A (2008). Sample size calculation for cluster randomized cross-over trials. Stat Med.

[63] Hussey MA, Hughes JP (2007). Design and analysis of stepped wedge cluster randomized trials. Contemp Clin Trials.

