# Supplementary figures and images for: Intra-cluster and inter-period correlation coefficients for cross-sectional cluster randomised controlled trials for type-2 diabetes in UK primary care
Source: Trials. 2016 Aug 15;17:402. doi: 10.1186/s13063-016-1532-9 (PMC4983799; doi:10.1186/s13063-016-1532-9)

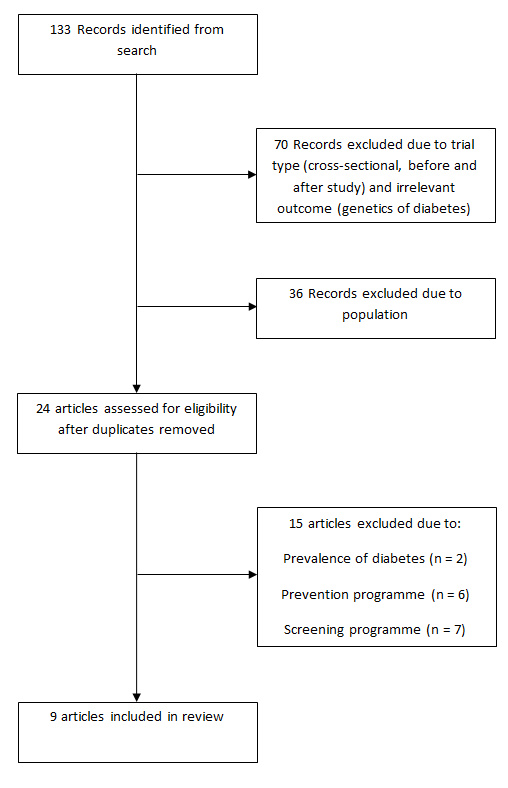

Supplement: Additional file 1: — Flow diagram of included trials for systematic search of trials undertaken in primary care in type-2 diabetes. (PNG 17 kb) [file 13063_2016_1532_MOESM1_ESM.png]
